# Keratin and S100 calcium-binding proteins are major constituents of the bovine teat canal lining

**DOI:** 10.1186/s13567-015-0227-7

**Published:** 2015-09-25

**Authors:** Grant A. Smolenski, Ray T. Cursons, Brad C. Hine, Thomas T. Wheeler

**Affiliations:** Dairy Foods, AgResearch Ltd, Ruakura Research Centre, Hamilton, 3240 New Zealand; University of Waikato, Hamilton, 3240 New Zealand; CSIRO, Agriculture Flagship, Chiswick, Armidale, NSW 2350 Australia

## Abstract

The bovine teat canal provides the first-line of defence against pathogenic bacteria infecting the mammary gland, yet the protein composition and host-defence functionality of the teat canal lining (TCL) are not well characterised. In this study, TCL collected from six healthy lactating dairy cows was subjected to two-dimensional electrophoresis (2-DE) and mass spectrometry. The abundance and location of selected identified proteins were determined by western blotting and fluorescence immunohistochemistry. The variability of abundance among individual cows was also investigated. Two dominant clusters of proteins were detected in the TCL, comprising members of the keratin and S100 families of proteins. The S100 proteins were localised to the teat canal keratinocytes and were particularly predominant in the cornified outermost layer of the teat canal epithelium. Significant between-animal variation in the abundance of the S100 proteins in the TCL was demonstrated. Four of the six identified S100 proteins have been reported to have antimicrobial activity, suggesting that the TCL has additional functionality beyond being a physical barrier to invading microorganisms. These findings provide new insights into understanding host-defence of the teat canal and resistance of cows to mastitis.

## Introduction

Mastitis is a significant economic and welfare issue facing the dairy industry worldwide [1]. Susceptibility of cows to infection by mastitis-causing microorganisms varies considerably amongst dairy cattle [2] but the factors contributing to this variation are poorly understood. The teat canal is the first host tissue that bacteria encounter on the way to colonising the mammary gland. Inoculation of pathogens directly into the teat sinus, bypassing the teat canal, significantly increases the likelihood of developing a clinical infection compared to inoculation within the teat canal [3]. One interpretation of this finding is that the teat canal has a host-defence capacity and conceivably, this could influence susceptibility to mastitis.

Knowledge is limited regarding the specific host-defence mechanisms operating in the teat canal. However, the expression of key innate immune effector molecules and the phenotype of activated macrophages in the teat end tissues have recently been reported by several research groups [4-7]. The teat canal is lined with a sebum-like material and a number of studies [8-10] have demonstrated growth inhibitory activity against mastitis-causing bacteria by lipids derived from this teat canal lining (TCL). Cationic proteins have been enriched from the TCL and been shown to inhibit the growth of mastitis-causing bacteria [11,12]. However, to date the identity of these cationic proteins is still to be established, and the detailed protein composition of the TCL has not been characterised.

The skin epithelium has a similar morphology to that of the teat canal and also has a host-defence function [13]. Human skin epithelium is known to contain a number of antimicrobial proteins including lactoferrin, lysozyme, cathelicidin, RNase7, S100A7 and several β-defensin proteins [14]. These proteins have been shown to suppress the growth of bacteria and contribute to the host-defence function of skin [15,16]. It is, therefore, reasonable to postulate that the teat canal epithelium also produces antimicrobial proteins.

Modern protein separation and identification technologies allow a much more detailed characterisation of the TCL proteins than has been previously reported. In an attempt to better understand the host-defence capacity of the teat canal we characterised the protein composition of the TCL collected from healthy lactating dairy cows. Proteins isolated from the TCL were subjected to two-dimensional gel electrophoresis (2-DE) and mass spectrometry (MS) in order to profile the TCL proteome, identifying in particular those proteins associated with host-defence.

## Materials and methods

### Cows and collection of tissue

The experimental procedures involving cows used in this study were approved by the Ruakura and the University of Waikato Animal Ethics Committees acting in accordance with the guidelines of the New Zealand Animal Welfare Act 1999. Six Holstein-Friesian x Jersey crossbred dairy cows in late lactation (257 ± 30 days in milk) were enrolled in the study. The cows ranged from 3 to 7 years of age, average daily milk yield was 13.4 ± 2.2 L, and were of mixed gestation status. The average length of the hindquarter (8.9 ± 0.4 mm) and forequarter (8.7 ± 0.4 mm) teat canals were not significantly different in this group of cows. All six cows had a quarter milk somatic cell count (SCC) of less than 200 000 cells/mL, and were confirmed to be free of infections of the udder by bacterial culture of four successive aseptically collected foremilk samples over a three week period prior to the trial. Bacterial examination was performed under aerobic conditions by streaking 10 μL of each quarter milk sample onto an esculin blood agar plate and incubating for 24 h to 48 h at 37 °C. Mammary quarters were considered to be infected when six or more individual colonies of a bacterial species were cultured from the quarter milk. One of the 24 quarters was found to be infected with *Streptococcus uberis* and was, therefore, excluded from the study. The SCC of the 23 remaining uninfected quarters from the final milking ranged from 13 000 to 198 000 cells/mL, with an average of 72 500 cells/mL. The cows were slaughtered and two contralateral teats consisting of a forequarter and hindquarter teat were collected from each of the six cows. The teats were then dissected along the sagittal plane from the teat sinus through the teat canal to the teat orifice. Excess tissue was removed, and a segment of teat canal tissue was immediately frozen in liquid nitrogen for histological processing. For 2-DE and western analysis, teat canals from the remaining 11 teats were sliced open to expose the length of the teat canal. The sebum-like material present on the inner surface of the teat canal was removed by gently scraping a clean scalpel blade over the whole surface of the canal, resulting in an accumulation of the lining material on the blade. After removal of the TCL samples, the teats were snap-frozen in liquid nitrogen to solidify the teat tissue. Slivers of the frozen epithelial layer of the outer surface of the teat were cut using a clean scalpel blade to provide samples of skin epithelium. The TCL and teat skin were transferred to pre-weighed 1.5 mL tubes and immediately frozen in liquid nitrogen before being stored at −80 °C for subsequent analysis.

### Two-dimensional gel electrophoresis (2-DE)

Each TCL and skin sample was weighed and resuspended in 2D lysis buffer (50 mM Tris, pH 8.0; 8 M urea, 3 M thiourea, 65 mM DTT, 4% CHAPS, 0.1% SDS) to achieve a final tissue:buffer ratio of 25 mg of sample (wet weight)/mL of lysis buffer. The samples were shaken vigorously for 1 h at room temperature and incubated in a sonicating water bath for 15 min. After a second round of shaking and sonication the lysates were then centrifuged for 15 min at 14 000 × *g* to sediment any insoluble material. The protein concentration of the supernatant was determined using a 2-D Quant protein assay kit (GE Healthcare, NZ), using bovine serum albumin as the protein standard. An equal amount of protein from each of the six cows was then pooled in order to create a sample with enough protein for 2-DE analysis, which was performed as previously described [17]. A 350 μg portion of the pooled TCL extract as well as the pooled teat skin extract was subjected to isoelectric focusing using 18 cm, pH 3–11, non-linear, immobilised pH gradient strips for a total of 95 kVh. These proteins were then resolved in the second dimension by SDS electrophoresis using 14% polyacrylamide gels. Each sample was analysed in triplicate.

The 2-DE gels were stained for protein using colloidal Coomassie G-250 and then scanned using a GS-800 densitometer (Bio-Rad, Hercules, CA, USA). The images were processed using the Quantity One (v4.6.6) 1-D Analysis software package (Bio-Rad). Spot matching and quantification was performed using the PDQuest (v8.0.1) Advanced 2-D analysis software package (Bio-Rad).

### In-gel enzymatic digestion

Approximately 1 mm^2^ pieces of gel were excised for each 2-DE spot selected for analysis and processed for MS as previously described [18]. Briefly, the Coomassie stain was removed from each gel piece, the proteins were digested with trypsin and the tryptic peptides were extracted and lyophilised. The dried peptide extracts were reconstituted in 50 μL of 2 (v/v) acetonitrile in 0.2% (v/v) formic acid for subsequent MS analysis.

### Protein identification by tandem mass spectrometry

The protein digests were analysed by MS using a nano-Advance UHPLC (Bruker-Daltonics, Bremen, Germany) coupled to a maXis Impact ultra-high resolution quadrupole time-of-flight mass spectrometer (Bruker-Daltonics). For each sample, 5 μL was loaded onto a Magic C18AQ nano trap column (Bruker-Michrom, Bremen, Germany) in mobile phase buffer A, which comprised 0.1% (v/v) formic acid in water, at a flow rate of 5000 nL/min for 5 min. The trap column was switched in-line with an Intensity C18P analytical column (Bruker-Michrom) heated to 50 °C. Peptides were eluted from the trap column to the analytical column using a gradient of mobile phase buffer A and buffer B (0.1% (v/v) formic acid in 100% acetonitrile) increasing from 2% B to 45% B over 25 min at a flow rate of 800 nL/min. The eluate from the analytical column was directed to the captive spray ionisation source (Bruker-Daltonics), and mass spectra were generated using data-dependent analysis mode with dynamic exclusion set to 0.2 min. The three most abundant precursor ions were automatically chosen for fragmentation during each MS scan in the mass range of 300–1250 m/z. Mass spectra were deconvoluted using the Compass Data Analysis (v4.0 SP5) software package (Bruker Daltonics) and peak lists and imported into the ProteinScape (v3.1.3) software package (Bruker Daltonics) for data analysis. Peak lists were queried against the *Bos taurus* NCBI non-redundant database (release date, Jan. 22, 2014) using the Mascot (v2.3) search engine (Matrix Science, London, UK). For protein identification, the MS and MS/MS error tolerances were set to ±20 ppm and ±0.05 Da, respectively, and one missed tryptic cleavage was allowed. Carbamidomethylation of cysteine was included as a fixed modification and N-terminal ammonia-loss, deamidation of asparagine and glutamine, phosphorylation of serine and threonine, and oxidation of methionine were set as variable modifications. Search results were compiled and analysed by ProteinScape and peptide identifications were accepted if the Mascot ion score was ≥25 using a significance threshold of *p* < 0.05.

### Antibodies

Antiserum directed against full-length recombinant bovine S100A7 was a kind gift from Prof. H. Sauerwein and was produced as previously described [19]. Polyclonal antibodies directed against peptide-keyhole limpet hemocyanin conjugates were raised in New Zealand White rabbits using established methods [20]. The peptides used as immunogens were C-MLTDLECAINS (amino acids 1–11 of bovine S100A8); C-MEDKMSQMESSIETI (amino acids 1–15 of bovine S100A9) and C-VSRVLKTAHIDIHKE (amino acids 78–92 of bovine S100A12). Immunoglobulin was purified from the antiserum by protein-A affinity chromatography.

### Western blotting

The TCL protein extracts in 2D lysis buffer were diluted four-fold in a buffer containing 62.5 mM Tris (pH 6.8), 5% (v/v) glycerol, 5% (v/v) β-mercaptoethanol, 2% (w/v) SDS and resolved by SDS-PAGE on 12% Bis-Tris XT criterion gels (Bio-Rad) using 2-(N-morpholino) ethanesulfonic acid (MES) running buffer. The total amount of TCL extract loaded was 0.8 μg, 10 μg, 10 μg, and 4 μg for detection of S100A7, S100A8, S100A9, and S100A12, respectively. The resolved proteins were transferred onto reinforced nitrocellulose membranes using an iBlot Gel Transfer System (GE Healthcare), according to the manufacturer’s instructions. The membranes were blocked with 4% (w/v) skim milk powder made up in Tris-buffered saline (10 mM Tris, pH 7.5; 150 mM NaCl, pH 7.5) (TBS), containing 0.1% bovine serum albumin and 0.1% Tween-20 (TBS/BSA/T) for 2 h and then probed for 16 h at 4 °C with TBS/BSA/T containing 0.12 μg/mL of S100A7 antiserum, or 1.5 μg/mL of purified immunoglobulin directed against S100A8, S100A9, or S100A12. The membranes were washed with TBS and reprobed for 2 h with 50 ng/mL goat anti-rabbit immunoglobulin conjugated to horseradish peroxidase (Sigma-Aldrich, St. Louis, MO, USA). The blots were visualised using enhanced chemiluminescence as previously described [21]. The signal was captured using a ChemiDoc XRS (Bio-Rad) as well as XAR X-ray film (Kodak, Rochester, NY, USA) and the band intensities were quantified using Quantity One (Bio-Rad). The relative abundance of each protein was determined on individual membranes by reference to a four-point standard curve produced from serial 2-fold dilutions of a positive control sample (teat skin for S100A7; neutrophil lysate for S100A8, S100A9, and S100A12) run on the same gel. An equivalently loaded gel was stained with colloidal Coomassie blue and subjected to densitometry to confirm equal protein loading as previously described [22].

### Immunofluorescence

Tissues were thawed to −5 °C and cut into transverse sections so as to expose a cross section of the teat canal at its mid-point, approximately 4–5 mm proximal to the teat canal orifice. Each section was embedded in 1 cm diameter moulds with Tissue-Tek O.C.T. compound (Sakura Finetek, CA, USA) and stored at −80 °C. Serial 5 μm thick cryosections of the teat canal were then cut from this frozen embedded section with a microtome. Each slice was mounted on polysine glass microscope slides (Thermo Fisher Scientific, USA) and the tissue dried overnight at room temperature. The slides were then fixed for 8 min in ice-cold ethanol, washed twice in TBS containing 0.1% Tween-20 (TBST) and blocked for 2 h at room temperature with 10% (v/v) goat serum in TBST. Anti-S100 immunoglobulin solutions were prepared in antibody amplifying buffer (StressMarq Biosciences, Canada) containing 3% (v/v) goat serum. The S100A7 antiserum was diluted to 18 μg/mL while the S100A8, S100A9 and S100A12 antibodies were each diluted to 150 μg/mL. The antibody solutions were overlaid onto one of two serial tissue sections on each slide and an equivalent concentration of immunoglobulin from a non-immunised rabbit was applied to the remaining section to act as a negative control. Sections were incubated overnight in a humidified chamber at 4 °C. The slides were then washed with TBST and incubated in 1 μg/mL of Alexa Fluor 594-conjugated goat anti-rabbit secondary antibody (Life Technologies, CA, USA) for 1 h, at room temperature. The slides were then washed again with TBST and counter-stained with 0.4 μM diamidinophenylindole hydrochloride (DAPI) in TBS to visualise the nuclei. Tissue sections were mounted with fluorescence mounting medium (Dako, CA, USA) and then photographed using a Leica DMI 6000B inverted fluorescence microscope (Leica Microsystems, CMS, GmbH) equipped with a DFC300 FX digital colour CCD camera (Leica Microsystems). Alexa Fluor 594 signal was detected using a Leica TX2 filter cube (excitation: BP 560/40, emission: BP 645/75). Images from each section were adjusted to match for contrast and brightness using Coral Paint Shop Pro Photo XI. Both the DAPI and Alexa Fluor images of each tissue slice were merged together using SPOT advance software (v4.6.4.3; Diagnostic Instruments, Sterling Heights, MI, USA).

### Statistical analysis

All values are expressed as mean values ± standard error of the mean (SEM). Differences between the abundance of TCL S100 proteins were assessed using the unpaired two-tailed Students *t* test. Restricted Maximum Likelihood (REML) analysis was performed using Genstat 16 (VSN International, Hemel Hempstead, UK) to estimate the within—and between-animal variation of S100 proteins. The *p*-values for REML analysis were derived using the modified *F*-statistic and the threshold level for statistical significance was set at *p* < 0.05.

## Results

### Keratins and S100 proteins constitute the major components of the teat canal lining

**Figure 1:**
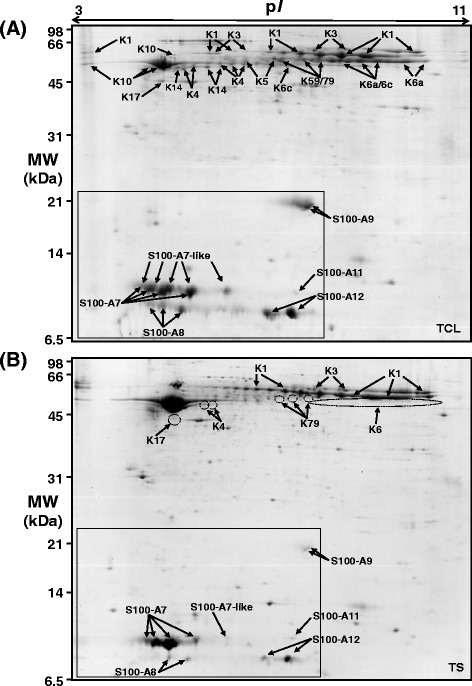
**2-DE proteome map of bovine teat canal lining and teat skin.** (**A**) Pooled TCL proteins (350 μg) were subjected to 2-DE and detected by colloidal Coomassie blue staining. The relative molecular weight (MW) is given on the left, while the p*I* range is given at the top of the figure. Thirty six protein spots corresponding to identified keratin proteins and 17 S100 protein spots (enclosed in the box) are indicated by arrows. Keratin abbreviations are (K1) keratin, type II, cytoskeletal 1; (K3) keratin, type II, cytoskeletal 3; (K4) keratin, type II, cytoskeletal 4; (K5) keratin, type II, cytoskeletal 5; (K6a) keratin, type II, cytoskeletal 6A; (K6c) keratin, type II, cytoskeletal 6C; (K10) keratin, type I, cytoskeletal 10; (K14) keratin, type I, cytoskeletal 14; (K17) keratin, type I, cytoskeletal 17; (K59) keratin, type II, cytoskeletal 59, component IV; (K79) keratin, type II, cytoskeletal 79. (**B**) Pooled teat skin proteins (350 μg) were subjected to 2-DE and detected by colloidal Coomassie blue staining. The absence or lowered relative abundance of keratin proteins, compared to TCL, is indicated by dotted circles. Keratin and S100 protein spots identified by MS are indicated by arrows. TCL, teat canal lining; TS, teat skin.

Analysis of pooled teat canal lining by 2-DE revealed a complex pattern of spots dominated by two clusters of proteins of relatively high abundance (Figure 1A). The main cluster contained many resolvable protein spots between 50 to 70 kDa ranging from p*I* 4.5 to 9.5. These were estimated to comprise 51% of the total resolvable protein by densitometry. A total of 36 of these spots were identified by MS as keratins (indicated by arrows labelled with a K in Figure 1A). Some of the complexity observed among the keratins is likely due to variable phosphorylation as has been previously reported to be the case for keratins in other epithelial tissues [23]. The primary (cell-type defining) keratins K1 [GenBank: XP_003584943], K3 [XP_003584947], K4 [NP_001091855], K5 [DAA30012], K10 [NP_776802] and K14 [NP_001160047] were identified as well as the secondary keratins K6a [NP_001076979], K6c [XP_884760], K17 [A1L595], K59 [NP_001244333] and K79 [NP_001068816]. Two different isoforms of keratin K6 were identified with the K6a isoform predominating over the K6c isoform. This mixture of keratins is consistent with proteins originating from hyper-proliferative stratified squamous epithelium.

A separate cluster of proteins between 10 to 12 kDa with a p*I* ranging from 4.5 to 6.5 (Spots arrowed within the box in Figure 1A) were also identified. Seventeen of these spots were identified by MS as belonging to six different members of the S100 family of calcium-binding proteins. These include S100A12 [NP_777076.1], S100A11 [DAA31649.1], S100A9 [NP_001039793.1], S100A8 [NP_001107197.1], S100A7 [NP_777021.1], and an S100A7-like protein [XP _875693.1] predicted from *Bos taurus* genome sequencing. In total, these seventeen S100 protein spots comprise 19% of the total resolvable protein by 2-DE as estimated by densitometry. As with the keratins, many of the S100 proteins were also present as trains of horizontal spots, suggesting that they are also post-translationally modified. The two most abundant S100 proteins identified were S100A7 and the S100A7-like protein, represented in the gels as trains of four and five spots, respectively.

Samples of skin taken from the outer surface of the teat were analysed by 2-DE and MS in order to compare the protein profile with that of the TCL. This revealed a number of differences in the protein profile of skin compared with the TCL profile (Figure 1B). For example, the relative abundance of the protein spots corresponding to K1, K3, K5, K10 and K14 is higher in teat skin compared with the TCL. On the other hand, there was a decrease in relative abundance of the K4, K6, K17 and K79 protein spots (areas indicated by dotted circles in Figure 1B). The difference in the K6 series of spots is particularly striking as it is one of the most abundant keratin proteins in the TCL but is present only at low abundance in teat skin. Densitometry analysis of each of the replicate gels revealed that the summed relative abundance of S100A7, S100A7-like, S100A8, S100A9, S100A11 and S100A12 proteins in the TCL was greater by 1.5-fold, 15-fold, 5.9-fold, 6.1-fold, 1.5-fold and 2.6-fold respectively than the summed relative abundance of the corresponding protein spots in the teat skin gels (S100 spots arrowed in Figure 1B). These differences were statistically significant (*p* < 0.05) between the two tissues for five of the six S100 proteins, the exception being S100A7.

### S100 proteins are concentrated in the cornified layer of the TCL

**Figure 2:**
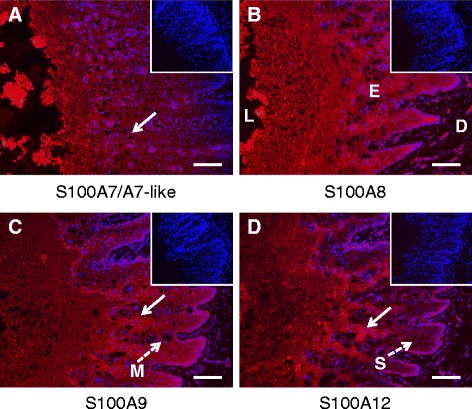
**Immunofluorescence of bovine teat canal tissue.** Teat canal cryosections were stained using indirect immunofluorescence for (**A**) S100A7/A7-like, (**B**) S100A8, (**C**) S100A9 and (**D**) S100A12. Serial sections were probed with anti-S100 protein antibodies and the signal detected with Alexa Fluor labelled secondary antibodies (red signal). Each section was counterstained with DAPI (blue signal). Equivalent sections probed with non-specific IgG are shown as inserts on the upper right hand corner. All micrographs were exposed for the same length of time as the negative controls. L: lumen, E: epidermis, D: dermis, M: Marksäulchen, S: *stratum spinosum*. Scale bars in all panels are 100 μm.

S100 proteins have been previously identified in normal mammalian skin [24] as well as in neutrophils isolated from human blood [25] and in milk from cows with mastitis [26]. To determine if the S100 proteins found in the TCL were locally produced by the teat canal epithelium, immunohistochemical analysis of teat canal sections was performed. Indirect immunofluorescence revealed strong signals in the epidermal layer of the teat canal epithelium for S100A7, S100A8, S100A9, and S100A12 (Figure 2). The presence of S100A7, S100A8, S100A9, and S100A12 proteins was detected in all 12 teats that were analysed and no obvious variations in signal intensity or localisation were observed among individual cows for any of the four S100 antibodies. Figure 2 is representative for all 12 teats analysed. The majority of the signal from all four S100 proteins was restricted to the epidermal layer in which keratinocytes are the predominant cell type. The signal was concentrated in the cornified layer of the epidermis, but distinct patterns of expression were observed for each of the S100 proteins. For example, S100A7 signal was observed predominantly within the stratum granulosum (see arrow in Figure 2A), whereas an intense signal for S100A8 and S100A9 was observed throughout the epithelial layer. The most concentrated signal for S100A9 and S100A12 was in the inter-papillar zones of the epithelium between the teat canal rete ridges (arrowed in Figures 2C and D). S100A12 was also present in all epidermal layers; however, the signal in the lower spinous layer (marked S in Figure 2D) was less intense than for S100A8 and S100A9. For S100A8, S100A9 and S100A12, signals were also observed in non-epithelial cells located directly beneath the basal cells of the epidermis and within the supra-papillary plate between the rete ridges (Figures 2B-D). Some signal was also evident in the non-epithelial cells inside the circular follicle-like structures that are present within the epidermal layer. These structures, which are not present in the skin epithelium, are known as Marksäulchen [27] (marked M in Figure 2C). Immunofluorescent signal for all four S100 proteins was also observed in the teat skin. For S100A7, the signal was as strong as in the teat canal epithelium whereas for S100A8, S100A9 and S100A12 the signal was noticeably less intense (results not shown).

### Between and within animal variability in the abundance of S100 proteins in the teat canal lining

**Figure 3:**
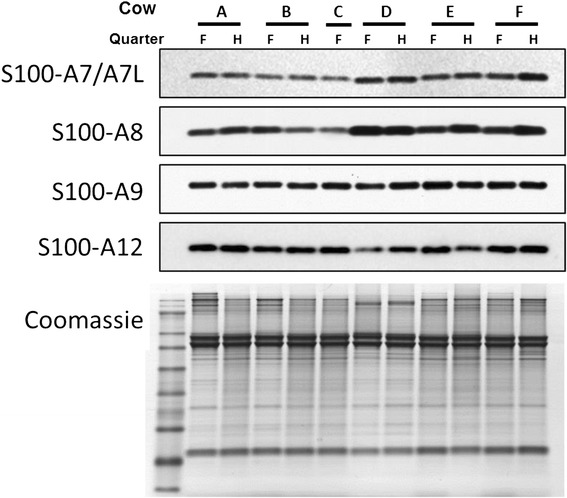
**Western blotting of teat canal lining from individual cows.** Proteins extracted from the TCL of six individual cows (A-F) were subjected to SDS–PAGE followed by western blot analysis. Samples were collected from a forequarter (F) and hindquarter (H) teat from each cow. The blots were probed with antibodies directed against bovine S100A7/A7-like, S100A8, S100A9, and S100A12. A replicate gel loaded with 10 μg of total protein was stained with Coomassie blue G-250 (bottom panel) to demonstrate equivalent loading.

**Figure 4:**
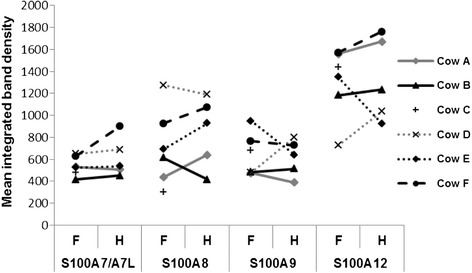
**Abundance of S100 proteins in teat canal lining from individual teats.** TCL proteins were subjected to quantitative western blotting using internal standards. Chemiluminescence signal was captured by a CCD camera and densitometry performed on the images. The average untransformed integrated density values for each S100 protein and teat are shown in the plot. S100 proteins measured from forequarter (F) and hindquarter (H) teats from the same cow are horizontally linked for each antibody.

The relative abundance of host-defence proteins in individual cows may be a factor influencing susceptibility to infection. Therefore, quantitative western blot analysis was performed in triplicate on each of the TCL samples from individual teats (one forequarter and one hindquarter teat from each individual cow) in order to assess the between versus within animal variability in abundance of the S100 proteins. The S100A7 and S100A7-like proteins were not assessed separately due to the unavailability of antibodies that recognise each separately. Representative images of three replicate western blot analyses are shown in Figure 3, and the mean abundance of each S100 protein, obtained after quantitative chemiluminescence analysis, is shown in Figure 4. Analysis of the replicates produced an overall coefficient of variation (CV) of less than 7%. This technical variation was too low to depict in Figure 4. Significant variability (above that expected due to technical variation) in the abundance of each of the S100 proteins investigated was observed. A 2.2-fold, 4.2-fold, 2.4-fold and 2.4-fold range in protein abundance between teats was detected for S100A7/A7-like, S100A8, S100A9 and S100A12 with coefficients of variation of 14.6%, 39%, 15.3% and 20%, respectively. The observed between teat variation in abundance of S100A8 was almost twice that of the other S100 proteins. Estimates of the within and between animal variation in S100 protein abundance produced were similar for S100A7/A7-like, S100A9 and S100A12. However, for S100A8 the observed between cow variation was significantly higher (*p* < 0.05) than the within cow variation (CV of 39% versus 24.8%, respectively) suggesting that the basal expression of this protein in the TCL differs more between cows than it does between teats within a cow.

## Discussion

To our knowledge, this is the first report of an analysis of the bovine TCL using 2-DE. Our results confirmed the long standing assumption that keratins are the predominant proteins in the TCL, but also revealed that multiple members of a family of S100 calcium-binding proteins are also present at relatively high abundance. The results show that multiple forms of both the keratins and the S100 proteins are present and that their protein profiles differ significantly between the TCL and the teat skin. Further research is required to distinguish the abundances of the S100A7 and S100A7-like proteins as these two proteins, which share 91% amino acid identity, were not able to be reliably distinguished from one another using currently available antibodies.

The relative abundance of the keratins in the 2-DE gels is consistent with the known physiology of the TCL. Multiple primary acidic and basic keratins typical of stratified squamous epithelia (K1, K3, K4, K5, K10, K14) are present as well as the secondary keratins (K6, K17) [28]. K6 and K17 are not normally expressed in healthy skin epithelium [29] and are only observed in this tissue during periods of physiological stress such as wound-healing [30], psoriasis [31] or in certain carcinomas [32] where epithelial cells are undergoing periods of rapid proliferation.

The presence of S100 proteins in the TCL is consistent with earlier reports of S100 proteins being identified in skin [33] albeit during periods of physiological stress. The teat canal is thought to develop as an invagination of the outer teat skin, accounting for the morphological similarities between the two tissues [34]. However, the distinct pattern of the abundance of the S100 proteins between the teat canal and the outer teat skin suggests that there are functional differences between them. There is evidence that some S100 proteins contribute to epithelial host defence. In human skin and bovine teat skin, S100A7 is highly effective in killing *Escherichia coli* and is moderately effective against other bacterial species [19,35]. Intracellular expression of the S100A8/A9 heterodimer complex calprotectin results in greater resistance to bacterial invasion by epithelial cells [36,37] as well as reducing the ability of fungi to infect epithelial cells of the oral mucosa [38]. Broad spectrum antimicrobial activity against a range of microorganisms has also been demonstrated for purified preparations of S100A7, S100A8, S100A9, and S100A12 at concentrations as low as 0.5 μmol/L [39-43]. These S100 family members have also been shown to be chemotactic to CD4^+^ lymphocytes, induce monocyte trafficking [44], and facilitate the recruitment of immune cells to the site of infection [45]. Calprotectin has been shown to interact with Toll-like receptor 4 (TLR4) [46], and all four S100 protein members are ligands for the receptor for advanced glycation end products (RAGE) [47,48] suggesting that they are also involved in pathogen recognition. Consistent with this, stimulation of mammary tissues with microbial pathogen-associated molecular patterns leads to the induction of S100 mRNAs [49]. Taken together, these observations suggest that the role of the S100 proteins in the TCL could be to limit the growth of microorganisms in the teat canal through multiple mechanisms; however the antimicrobial activity of S100 proteins isolated from the TCL was beyond the scope of the current study and not investigated here.

The distribution patterns of the S100 proteins observed in the TCL appear similar to the abundance and distribution patterns previously reported in human skin during the inflammatory condition psoriasis [33]. Also, as described above, the relatively high abundance of K6 and K17 in the TCL compared with normal skin is similar to that observed for skin undergoing repair or proliferation. These observations suggest that the teat canal epithelium is in a hyper-proliferative state during lactation, even in the absence of an infection. This high turnover of keratinocytes and consequent extensive cornification is likely to contribute to the physical barrier function of the teat canal as well as providing a biochemical barrier to infection.

In this study, we observed significant between-animal variation in the abundance of S100A8 expressed in the TCL. Since S100A8 has antimicrobial activity in the form of calprotectin, it is therefore conceivable that cows that produce lower amounts of S100A8 would be more susceptible to bacterial colonisation of the teat canal and consequently intra-mammary infections. Future studies are warranted to investigate associations between the basal expression of S100 proteins such as S100A8 in the TCL and susceptibility to mastitis infection.

The findings from this study provide new insights into understanding the host-defence capacity of the teat canal and the mechanisms influencing resistance to mastitis in dairy cows. This research supports the concept that the TCL has an active host-defence capacity as well as providing a physical barrier to infection of the mammary gland. The identification of S100 proteins in the TCL provides a focus for further work to confirm the involvement of specific molecules and pathways influencing susceptibility to mastitis.

## Abbreviations

TCL: Teat canal lining
2-DE: Two-dimensional gel electrophoresis
MS: Mass spectrometry
K: Keratin
